# Diabetes mellitus com controle glicêmico inadequado aumenta a mortalidade de adultos mais velhos brasileiros: evidências do ELSI-Brasil

**DOI:** 10.1590/0102-311XPT163525

**Published:** 2026-05-29

**Authors:** Luciana Lustosa Torres, Juliana Lustosa Torres, Amanda Cristina de Souza Andrade, Nair Tavares Milhem Ygnatios, Ariene Silva do Carmo, Maria Fernanda Lima-Costa, Larissa Loures Mendes

**Affiliations:** 1 Universidade Federal de Minas Gerais, Belo Horizonte, Brasil.; 2 Instituto René Rachou, Fundação Oswaldo Cruz, Belo Horizonte, Brasil.; 3 Núcleo de Estudos em Saúde Pública e Envelhecimento, Fundação Oswaldo Cruz/Universidade Federal de Minas Gerais, Belo Horizonte, Brasil.

**Keywords:** Diabetes Mellitus, Idoso, Controle Glicêmico, Mortalidade, Diabetes Mellitus, Aged, Glycemic Control, Mortality, Diabetes Mellitus, Anciano, Control Glucémico, Mortalidad

## Abstract

Este estudo teve por objetivo avaliar a associação entre o controle glicêmico do diabetes mellitus e a mortalidade por todas as causas, considerando as categorias “sem diabetes mellitus”, “diabetes mellitus com controle glicêmico adequado” e “diabetes mellitus com controle glicêmico inadequado”. Utilizaram-se dados da linha de base (2015/2016) do ELSI-Brasil, com amostra nacional representativa da população com 50 anos ou mais, vinculados ao Sistema de Informações sobre Mortalidade (SIM) no período compreendido entre 2015 e 2021. O diabetes mellitus foi identificado por autorrelato, e o controle glicêmico avaliado pelos níveis de hemoglobina glicada, considerando-se inadequados os valores ≥ 7%. A mortalidade foi verificada pelas declarações de óbito e *linkagem* probabilística com o SIM. A análise estatística considerou os parâmetros amostrais e baseou-se em modelos de regressão de Cox. Dos 2.338 participantes, 16% tinham diabetes mellitus; destes, 49,1% apresentaram controle inadequado. Em cinco anos de seguimento, 194 óbitos foram registrados. O risco de mortalidade ajustado foi maior apenas no grupo com diabetes mellitus e controle inadequado (HR = 2,68; IC95%: 1,08-6,65) em relação aos sem diabetes mellitus. Os achados reforçam a importância do controle glicêmico e do seu monitoramento contínuo na atenção primária à saúde.

## Abstract (en)

This study evaluated the association between blood sugar control of diabetes mellitus and all-cause mortality, considering the categories “no diabetes mellitus”, “diabetes mellitus with adequate blood sugar control”, and “diabetes mellitus with inadequate blood sugar control”. Data from the ELSI-Brazil baseline survey (2015/2016) were used, with a representative national sample of the population aged 50 years or older, linked to the Brazilian Mortality Information System (SIM, acronym in Portuguese) in the 2015-2021 period. Diabetes mellitus was identified by self-report, and blood sugar control was assessed by glycated hemoglobin levels, and values ≥ 7% were considered inadequate. Mortality was verified by death certificates and probabilistic linkage with SIM. The statistical analysis considered the sample parameters and was based on Cox regression models. Among 2,338 participants, 16% had diabetes mellitus; of these, 49.1% had inadequate control. In five years of follow-up, 194 deaths were recorded. The adjusted mortality risk was higher only in the group with diabetes mellitus and inadequate control (HR = 2.68; 95%CI: 1.08-6.65) in relation to those without diabetes mellitus. The findings reinforce the importance of blood sugar control and its continuous monitoring in primary health care.

## Abstract (es)

Este estudio evaluó la asociación entre el control glucémico en la diabetes mellitus y la mortalidad por todas las causas, considerando las categorías “sin diabetes mellitus”, “diabetes mellitus con control glucémico adecuado” y “ diabetes mellitus con control glucémico inadecuado”. Se utilizaron datos de la línea de base (2015/2016) del ELSI-Brasil, con una muestra nacional representativa de la población de 50 años o más, vinculados al Sistema de Información de Mortalidad (SIM) para el período comprendido entre el 2015 y el 2021. La diabetes mellitus se identificó mediante autoinforme y el control glucémico se evaluó mediante los niveles de hemoglobina glucosilada, considerándose valores ≥ 7% como inadecuados. La mortalidad se verificó mediante certificados de defunción y vinculación probabilística con el SIM. El análisis estadístico consideró los parámetros muestrales y se basó en modelos de regresión de Cox. Entre 2.338 participantes, el 16% tenía diabetes mellitus; de éstos, el 49,1% tenía un control inadecuado. Durante cinco años de seguimiento, se registraron 194 muertes. El riesgo ajustado de mortalidad fue mayor solo en el grupo con diabetes mellitus y control inadecuado (HR = 2,68; IC95%: 1,08-6,65) en relación a aquellos sin diabetes mellitus. Los hallazgos refuerzan la importancia del control glucémico y de su seguimiento continuo en atención primaria de salud.

## Introdução

O Brasil enfrenta um rápido processo de envelhecimento populacional, acompanhado pelo aumento das doenças crônicas não transmissíveis (DCNT), entre elas o diabetes mellitus ^1^. Globalmente, a prevalência de diabetes mellitus entre indivíduos com 55 anos ou mais aumentou de 18,1% em 2015 para 19,1% em 2019, atingindo 19,5% em 2023 ^2^ e há a projeção de aumentar, nas pessoas entre 65 e 99 anos, de 135,6 milhões em 2019 para 276,2 milhões em 2045 ^3^. No Brasil, em 2019, a prevalência de diabetes mellitus foi de 20,2% naqueles com 60 anos ou mais ^4^. Em números absolutos, considerando todas as faixas etárias, o país representa o quinto maior número de casos de diabetes mellitus do mundo ^5^, com estimativas de alcançar 23,2 milhões de brasileiros vivendo com diabetes mellitus em 2045 ^6^. 

O diabetes mellitus é caracterizado pela hiperglicemia persistente, resultante da deficiência na secreção ou na ação da insulina, ou de ambos os mecanismos ^7^ ^,^ ^8^. Trata-se do segundo principal fator de risco metabólico relacionado à mortalidade ^9^. O controle glicêmico, avaliado comumente por meio da hemoglobina glicada (HbA1c), é fundamental para reduzir a ocorrência de complicações e prevenir óbitos ^10^. Valores de HbA1c abaixo de 7% têm sido associados a menor risco de desfechos adversos, sendo essa a meta recomendada por diretrizes internacionais e nacionais ^11^ ^,^ ^12^. No entanto, dados nacionais de 2014-2015 mostraram que apenas 48,1% dos brasileiros com 18 anos ou mais e diagnosticados com diabetes mellitus apresentavam controle glicêmico adequado ^12^. 

Estudos internacionais demonstram que níveis elevados de HbA1c em pessoas idosas com diabetes mellitus estão associados a um maior risco de mortalidade por todas as causas, em comparação à população sem diabetes mellitus ^13^ ^,^ ^14^ ^,^ ^15^ ^,^ ^16^ ^,^ ^17^. No entanto, há escassez de dados longitudinais baseados em amostras populacionais representativas de pessoas mais velhas no país. 

A análise dessa associação em adultos mais velhos é particularmente relevante devido às especificidades dessa população. O envelhecimento está associado a alterações fisiológicas, como sarcopenia e comprometimento das funções renal e hepática, além de maior ocorrência de comorbidades, limitações funcionais e uso de múltiplos medicamentos ^18^. Além disso, pessoas mais velhas tendem a ter mais tempo de evolução do diabetes mellitus, o que pode intensificar os riscos de mortalidade ^16^ ^,^ ^19^.

Diante desse contexto, esse estudo tem como objetivo avaliar se o diabetes mellitus e o controle glicêmico inadequado estão associados a um maior risco de morte por todas as causas em adultos brasileiros com 50 anos ou mais, em uma amostra nacionalmente representativa dessa população. Pretende-se, por meio dos resultados, produzir evidências para subsidiar políticas públicas, tanto para o grupo sem diabetes mellitus, especialmente no âmbito da promoção à saúde, quanto para os grupos com diabetes mellitus, com controle glicêmico adequado e com controle glicêmico inadequado. Os achados podem orientar o planejamento de estratégias de cuidado e monitoramento direcionadas aos grupos de maior vulnerabilidade, contribuindo para a melhoria das políticas de controle do diabetes mellitus e à redução de seus impactos sobre a mortalidade no país.

## Métodos

### Fonte de dados

Este estudo foi baseado em dados da linha de base do *Estudo Longitudinal da Saúde dos Idosos Brasileiros* (ELSI-Brasil, 2015-2016) e a subsequente mortalidade dessa população em 5 anos. O ELSI-Brasil é um estudo de coorte de base populacional, nacionalmente representativo da população com 50 anos ou mais, realizado em 70 municípios ^20^ ^,^ ^21^. Sua amostragem utilizou um desenho com estágios de seleção, combinando estratificação de unidades primárias de amostragem (municípios), setores censitários e domicílios. Na linha de base, foram incluídos 9.412 participantes, dos quais 2.361 foram aleatoriamente subamostrados para a coleta de sangue, a fim de manter a representatividade nacional, e tornaram-se assim elegíveis para o presente trabalho. A coleta de sangue foi realizada no domicílio dos participantes por técnicos de laboratório previamente treinados. Todas as análises sanguíneas foram realizadas pelo mesmo laboratório, credenciado pelo Ministério da Saúde ^20^. 

A *linkagem* probabilística dos dados do SIM com os dados do ELSI-Brasil foi realizada pelo Secretaria de Vigilância em Saúde e Ambiente, Ministério da Saúde de forma restrita, através da técnica de filtros de Bloom ^22^, considerando o nome do participante, o sexo, a data de nascimento, o município de residência e o nome da mãe. Após aplicação da técnica, foram gerados escores variando entre 0 e 10.000, sendo considerados para análise os pares com escores maiores ou iguais a 9.000 pontos. Com base nessa *linkagem* foram identificados 609 óbitos (89,9% daqueles óbitos informados por um *proxy* dentre os participantes encontrados na onda 2 do ELSI-Brasil) e 263 óbitos (10,4% dos participantes perdidos durante o seguimento). Maiores detalhes sobre a *linkagem* dos dados podem ser consultados em publicações anteriores ^23^ ^,^ ^24^. 

Detalhes do estudo ELSI-Brasil podem ser vistos na *homepage* da pesquisa (https://elsi.cpqrr.fiocruz.br) e em publicações anteriores ^20^ ^,^ ^21^. O ELSI-Brasil foi aprovado pelo Comitê de Ética do Instituto René Rachou, Fundação Oswaldo Cruz (CAAE: 34649814.3.0000.5091) e todos os participantes assinaram Termos de Consentimento Livre e Esclarecido (TCLE) relacionados às entrevistas, às coletas de sangue e à *linkagem* com os dados do Sistema de Informações sobre Mortalidade (SIM) ^20^.

### Desfecho

O tempo até a morte por qualquer causa foi o desfecho principal do estudo. A data da entrevista na linha de base (maio de 2015 a outubro de 2016) foi considerada o tempo inicial do estudo, enquanto a data do óbito foi considerada como o tempo final da ocorrência do evento (falha) ou até a conclusão da segunda onda (março de 2021), nos casos que não foram a óbito ou perderam o seguimento (censura). Desse modo, o tempo médio de seguimento do estudo foi de cinco anos.

### Exposição principal

O controle do diabetes mellitus foi a variável independente principal obtida em duas etapas, utilizada para a classificação dos participantes em três categorias de análise: “sem diabetes mellitus”, “ diabetes mellitus com controle glicêmico adequado” e “diabetes mellitus com controle glicêmico inadequado”. Na primeira etapa, foi considerado o autorrelato do diagnóstico médico do diabetes mellitus por meio da pergunta: “Algum médico já lhe disse que o(a) Sr(a) tem diabetes (açúcar no sangue)?”. Aqueles participantes que responderam “não” foram alocados no grupo “sem diabetes mellitus”. Na segunda etapa, entre os participantes que responderam “sim”, foi utilizado o resultado do teste de HbA1c para a identificação do controle glicêmico adequado ou inadequado do diabetes mellitus, adotando-se como o ponto de corte o valor de 7% ^11^. Deste modo, quando o nível da HbA1c foi maior ou igual a 7%, o participante foi alocado no grupo do “ diabetes mellitus com controle glicêmico inadequado” e, quando o nível da HbA1c foi menor que 7%, o participante foi alocado no grupo do “ diabetes mellitus com controle glicêmico adequado”.

O exame de sangue da hemoglobina glicada (HbA1) foi realizado pelo método de cromatografia líquida de alta eficiência (*High Performance Liquid Chromatography*, HPLC), método-padrão do DCCT (*Estudo sobre Complicações Crônicas do Diabetes*, acrônimo em inglês) ^20^.

### Variáveis de ajuste

As variáveis de ajuste deste estudo foram definidas com base na literatura ^25^ ^,^ ^26^ ^,^ ^27^ ^,^ ^28^ ^,^ ^29^ ^,^ ^30^ ^,^ ^31^ ^,^ ^32^ e incluíram características sociodemográficas e relacionadas à saúde, classificadas da seguinte forma: (a) Características sociodemográficas: sexo (feminino ou masculino); idade (contínua); escolaridade, em anos completos de estudo (< 8 anos, 8-11 anos ou ≥ 12 anos); área de residência (urbana ou rural). (b) Características relacionadas à saúde: consumo de frutas e vegetais (adequado ou inadequado); tabagismo (não ou sim), considerando-se fumar atualmente ou já ter fumado diariamente no passado; consumo excessivo de álcool (não ou sim); prática de atividade física (suficiente ou insuficiente); circunferência da cintura (adequada ou elevada); doenças do coração (infarto do coração, angina do peito ou insuficiência cardíaca) (não ou sim); e, hipertensão arterial sistêmica (HAS) (não ou sim). 

O consumo de frutas e vegetais adequado foi definido de acordo recomendação da Organização Mundial da Saúde de 400g/dia ^25^ ou de cinco porções de 80g cada ^26^. Devido às dificuldades de se transmitir aos entrevistados da pesquisa o conceito de porções de alimentos, considerou-se porção equivalente a número de vezes ao dia ^27^. O consumo recomendado de frutas e vegetais, incluindo suco de fruta natural, foi definido como cinco ou mais porções ao dia em cinco ou mais dias da semana ^27^, determinado pela frequência diária e semanal de frutas, suco de fruta natural ou verdura ou legume (alface, tomate, couve, cenoura, chuchu, berinjela, abobrinha - não foram considerados batata, mandioca ou inhame).

O consumo excessivo de álcool foi avaliado por autorrelato e definido de acordo com as recomendações do Instituto Nacional sobre Abuso de Álcool e Alcoolismo dos Estados Unidos: sete ou mais doses por semana para mulheres e quatorze ou mais para homens ou quando, em uma única ocasião, mulheres tomaram quatro ou mais doses e homens cinco ou mais doses ^28^.

A prática de atividade física insuficiente foi definida de acordo com o guia de atividade física para a população brasileira que recomenda às pessoas idosas a realização de pelo menos 150 minutos de atividade física moderada ao longo da semana ou pelo menos 75 minutos de atividade vigorosa ^29^ e o nível de atividade física foi avaliada pelo *Questionário Internacional de Atividade Física* (IPAQ, acrônimo em inglês). em sua versão reduzida, traduzida e validada para o Brasil ^30^. Esse instrumento contém questões relacionadas à frequência (dias por semana) e à duração (tempo por dia) das atividades físicas (caminhada, atividades moderadas e atividades vigorosas) realizadas na semana anterior à entrevista, considerando apenas aquelas realizadas por pelo menos 10 minutos contínuos de cada vez ^30^.

A circunferência da cintura foi obtida com fita métrica no ponto médio entre a 10ª costela e a borda da crista ilíaca. Ela foi considerada elevada quando estava ≥ 80cm para mulheres de até 60 anos, ≥ 88,7cm para as de 60 anos ou mais, e ≥ 94cm para homens de até 60 anos e ≥ 96cm para homens acima de 60 anos ^31^ ^,^ ^32^.

As doenças do coração e a hipertensão arterial sistêmica (HAS) foram avaliadas pelo diagnóstico médico autorrelatado. As doenças do coração incluíram infarto do coração, angina do peito ou insuficiência cardíaca.

Entre as pessoas que relataram ter diabetes mellitus, foram consideradas, descritivamente, o autorrelato de: “Alguma vez deixou de tomar algum do(s) remédio(s) para o diabetes?” (“sim” ou “não”), “Alguma vez diminuiu ou aumentou o número de comprimidos de algum do(s) remédio(s) para o diabetes?” (“sim” ou “não”), “Atualmente, por causa do diabetes, o(a) Sr(a) faz dieta?” (“sim” ou “não”), “Atualmente, por causa do diabetes, o(a) Sr(a) pratica atividade física?” (“sim” ou “não”), além do tempo de diagnóstico do diabetes mellitus (em anos). Todas foram avaliadas por meio do autorrelato.

### Análise estatística

Primeiramente, foi realizada a descrição das variáveis sociodemográficas e relacionadas à saúde para a amostra total, assim como de acordo com a mortalidade. As diferenças entre os grupos de mortalidade foram realizadas por meio do teste qui-quadrado de Pearson com correção de Rao Scott para variáveis categóricas e do teste t para variáveis contínuas. Das pessoas que já tinham o diagnóstico de diabetes mellitus, foi feita, adicionalmente, a descrição das variáveis relacionadas ao diabetes mellitus, verificando-se a diferença entre os grupos “diabetes mellitus com controle glicêmico adequado” e “diabetes mellitus com controle glicêmico inadequado”. A fim de verificar o risco de morte entre os grupos de diabetes mellitus analisados, foram utilizados modelos de regressão de Cox para estimar o *hazard ratio* (HR) e seus intervalos de 95% de confiança (IC95%). Todas as variáveis foram introduzidas no modelo ajustado, em modelos sequenciais de variáveis sociodemográficas e relacionadas à saúde. Foi adotado o nível de 5% de significância para todas as análises. A suposição dos riscos proporcionais foi verificada por meio dos resíduos de Schoenfeld. Baseado no modelo final, foi plotada a probabilidade de sobrevida em cinco anos, de acordo com o controle glicêmico. 

Os dados foram analisados utilizando-se o software Stata/SE versão 17.0 (https://www.stata.com), considerando o delineamento de amostra complexa e os pesos dos indivíduos subamostrados para a coleta de sangue, com o objetivo de manter a representatividade amostral.

## Resultados

**Figura 1:**
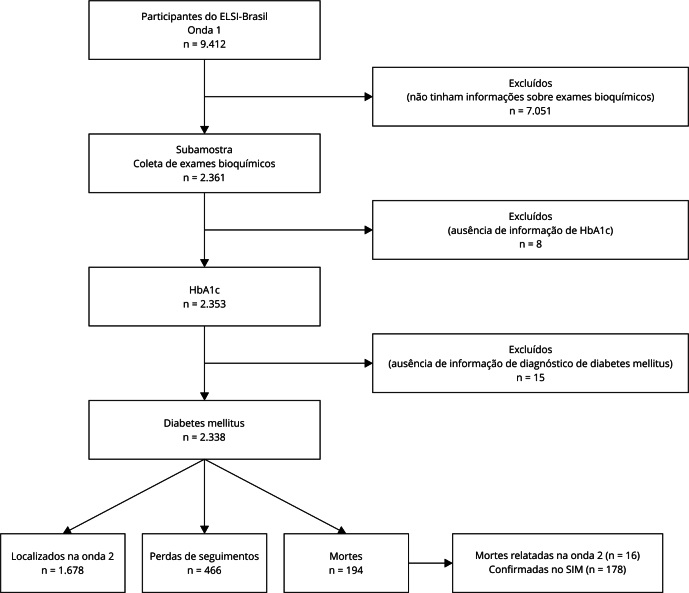
Fluxograma amostra analítica, *Estudo Longitudinal da Saúde dos Idosos Brasileiros* (ELSI-Brasil) 2015-2021.

HbA1c: Hemoglobina glicada; SIM: Sistema de Informações sobre Mortalidade. Nota: n = número de entrevistados, não incluindo correções de acordo com os parâmetros de amostragem.

A Figura 1 mostra o fluxograma dos participantes incluídos. Dos 2.361 participantes que realizaram a coleta de sangue, 8 foram perdas por ausência de informação do nível da hemoglobina glicada e 15 por ausência de informação acerca do relato de diagnóstico médico de diabetes mellitus, perfazendo uma amostra final de 2.338 participantes (99%). Até março de 2021, ocorreram 194 mortes (7,2%, das quais 178 foram confirmadas pela *linkagem* com o SIM), totalizando uma taxa de mortalidade de 14,0 por 1.000 pessoas/ano. O tempo médio de seguimento foi de 5,2 anos (±0,74). 

A Tabela 1 fornece estatísticas descritivas dos participantes. A média de idade dos participantes foi de 62,6 anos (desvio padrão 9,7 anos), com maior proporção de pessoas do sexo feminino (53,9%) e de baixa escolaridade (< 8 anos: 61,1%). Dentre os participantes, 84% não tinham diabetes mellitus, 8,2% tinham diabetes mellitus com controle glicêmico adequado e 7,8% tinham diabetes mellitus com controle glicêmico inadequado. Esses grupos foram diferentes em relação à proporção de mortalidade (p < 0,05), com as maiores taxas de mortalidade nos grupos de diabetes mellitus com controle glicêmico adequado e de diabetes mellitus com controle glicêmico inadequado, 24,4 e 27,5 por 1.000 pessoas/ano, respectivamente. As variáveis relacionadas à saúde que tiveram proporção de morte diferente entre os grupos foram o consumo excessivo de álcool, a prática de atividade física, a presença de doenças do coração e a HAS (p < 0,05). 

**Tabela 1:** Caracterização dos participantes em relação ao controle do diabetes mellitus, características sociodemográficas e estilo de vida, total e segundo a mortalidade. *Estudo Longitudinal da Saúde dos Idosos Brasileiros* (ELSI-Brasil), 2015-2021.

Variáveis	Total [n = 2.338]	Taxa de mortalidade (por 1.000 pessoas/ano)	Mortalidade		Valor de p
			Não (%) [n = 2.144]	Sim (%) [n = 194]	
Exposição principal					
Controle do diabetes mellitus					0,011
Sem diabetes mellitus	84,0	11,8	93,9	6,1	
Diabetes mellitus com controle glicêmico adequado	8,2	24,4	88,0	12,0	
Diabetes mellitus com controle glicêmico inadequado	7,8	27,5	86,0	14,0	
Variáveis de ajuste					
Sexo					< 0,001
Feminino	53,9	9,2	95,2	4,8	
Masculino	46,1	19,9	90,0	10,0	
Média de idade (DP)	62,6 (9,7)	-	62,1 (9,2)	70,0 (13,0)	< 0,001
Escolaridade (em anos completos)					0,011
< 8	61,1	17,7	90,9	9,1	
8 a 11	30,2	8,0	95,8	4,2	
≥ 12	8,7	7,9	95,8	4,2	
Área de residência					0,017
Urbana	89,9	13,2	93,2	6,8	
Rural	10,1	21,2	88,9	11,1	
Consumo recomendado de frutas e vegetais					0,495
Adequado	18,5	11,6	94,0	6,0	
Inadequado	81,5	14,0	92,8	7,2	
Tabagismo					0,170
Não	85,4	12,9	93,3	6,7	
Sim	14,6	20,8	89,7	10,3	
Consumo excessivo de álcool					0,019
Não	93,7	13,0	93,2	6,8	
Sim	6,3	30,1	85,4	14,6	
Prática de atividade física					< 0,001
Suficiente	69,9	9,9	94,9	5,1	
Insuficiente	30,1	24,4	87,7	12,3	
Circunferência da cintura					0,964
Adequada	36,2	11,6	93,9	6,1	
Elevada	63,8	11,8	93,9	6,1	
Doenças do coração					0,004
Não	85,2	12,4	93,5	6,5	
Sim	14,8	23,6	88,3	11,7	
HAS					0,006
Não	46,7	10,1	94,8	5,2	
Sim	53,3	17,5	91,0	9,0	

HAS: hipertensão arterial sistêmica; DP: desvio padrão.

Nota: %: considerando os parâmetros amostrais. n: sem considerar os parâmetros amostrais.

* Teste qui-quadrado de Pearson com correção de Rao-Scott.

A Tabela 2 mostra características relacionadas ao diabetes mellitus. O grupo diabetes mellitus com controle glicêmico inadequado relatou fazer mais atividade física como terapia para o diabetes mellitus (39,2%) e um maior tempo médio de diagnóstico (10,3 anos, desvio padrão 8,8). As demais características relacionadas ao diabetes mellitus e a causa básica do óbito não diferiram entre os grupos.

**Tabela 2:** Caracterização das variáveis relacionadas ao diabetes mellitus e causa básica do óbito entre os participantes com diagnóstico de diabetes mellitus. *Estudo Longitudinal da Saúde dos Idosos Brasileiros* (ELSI-Brasil), 2015-2021.

Variáveis	Diabetes mellitus com controle glicêmico adequado [n = 195]	Diabetes mellitus com controle glicêmico inadequado [n = 212]	Valor de p *
	% (n)	% (n)	
Relacionadas ao diabetes mellitus			
Deixar de tomar medicamento prescrito para diabetes mellitus nos últimos 7 dias	8,1 (22)	11,2 (20)	0,566
Diminuir ou aumentar o número de comprimidos prescritos para diabetes mellitus nos últimos 7 dias	5,7 (15)	7,5 (18)	0,494
Fazer dieta como terapia para diabetes mellitus	69,1 (138)	75,1 (154)	0,425
Fazer atividade física como terapia para diabetes mellitus	24,8 (55)	39,2 (70)	0,048
Tempo médio de diagnóstico de diabetes mellitus em anos (DP) **	6,4 (7,4)	10,3 (8,8)	0,002
Causa básica do óbito			
Doenças do aparelho circulatório	8,4 (3)	15,4 (7)	0,480
Doenças endócrinas, nutricionais e metabólicas	14,5 (7)	6,4 (5)	0,305

DP: desvio padrão.

Nota: %: considerando os parâmetros amostrais; n: sem considerar os parâmetros amostrais.

* Teste qui-quadrado de Pearson com correção de Rao-Scott para variáveis categóricas e teste t para a variável contínua;

** Variável contínua, descrita em média e DP.

A Tabela 3 apresenta resultados dos modelos brutos e ajustados de regressão de Cox. No modelo ajustado, a variável circunferência da cintura violou a suposição dos riscos proporcionais. Testou-se, portanto, a inclusão da variável contínua. Apesar de apresentar um resultado satisfatório no teste global (p = 0,405), o teste detalhado ainda mostrou uma violação dos riscos proporcionais da variável (p = 0,040). Como a circunferência da cintura é uma variável importante para o controle glicêmico, optou-se por colocar a circunferência da cintura como variável de estrato. Deste modo, o teste global mostrou-se satisfatório (p = 0,367) para riscos proporcionais. Testou-se, também, a inclusão das variáveis de atividade física (minutos em atividade física) e consumo de álcool (número de doses por ocasião) no modelo, sem utilizar pontos de corte, mas os resultados se mantiveram em relação aos modelos ajustados mostrados na Tabela 3. Deste modo, no modelo ajustado por todas as covariáveis e estratificado pela circunferência da cintura, as covariáveis sexo, idade, consumo excessivo de álcool e presença de doenças do coração associaram-se diretamente à mortalidade. O grupo diabetes mellitus com controle glicêmico adequado não apresentou maior risco de morte em relação ao grupo sem diabetes mellitus (HR = 1,86; IC95%: 0,95-3,64). Entretanto, encontrou-se uma associação diretamente proporcional do diabetes mellitus com controle glicêmico inadequado em relação à mortalidade por todas as causas (HR = 2,68; IC95%: 1,08-6,65). 

**Tabela 3:** Resultados dos modelos brutos e ajustados da associação entre controle do diabetes mellitus e mortalidade. *Estudo Longitudinal da Saúde dos Idosos Brasileiros* (ELSI-Brasil), 2015-2021.

Variáveis	Modelos brutos		Modelos ajustados			
			Modelo Cox *		Modelo Cox estratificado **	
	HR	IC95%	HR	IC95%	HR	IC95%
Exposição principal						
Controle do diabetes mellitus [*vs.* sem diabetes mellitus]						
Diabetes mellitus com controle glicêmico adequado	2,11	1,20-3,70	1,71	0,89-3,31	1,86	0,95-3,64
Diabetes mellitus com controle glicêmico inadequado	2,32	1,11-4,86	2,20	1,07-4,52	2,68	1,08-6,65
Variáveis de ajuste						
Sexo						
Masculino [*vs.* feminino]	2,18	1,55-3,08	2,24	1,49-3,36	2,12	1,41-3,19
Idade	1,07	1,05-1,09	1,06	1,04-1,09	1,08	1,06-1,10
Escolaridade (anos) [*vs.* < 8 anos]						
8 a 11	0,45	0,25-0,83	0,65	0,31-1,36	0,59	0,28-1,22
≥ 12	0,44	0,16-1,21	0,87	0,29-2,60	0,55	0,21-1,43
Área de residência rural [*vs.* urbana]	1,62	1,08-2,45	1,21	0,73-1,98	1,29	0,88-1,90
Consumo inadequado de frutas e vegetais [*vs*. adequado]	1,22	0,70-2,12	1,11	0,58-2,13	1,12	0,62-2,06
Tabagismo [*vs.* não]	1,63	0,84-3,16	1,90	1,01-3,38	1,60	0,80-3,22
Consumo excessivo de álcool (*vs.* não)	2,32	1,13-4,79	3,33	1,44-7,71	4,62	2,25-9,48
Prática de atividade física insuficiente (*vs. s*uficiente)	2,47	1,43-4,25	1,60	0,95-2,69	1,22	0,72-2,08
Doenças do coração (*vs.* não)	1,93	1,27-2,93	1,62	0,89-2,95	1,89	1,13-3,17
HAS (*vs.* não)	1,76	1,19-2,63	1,64	0,98-2,74	1,41	0,89-2,22

HAS: hipertensão arterial sistêmica; HR: *hazard ratio*; IC95%: intervalo de 95% de confiança.

Nota: em negrito: p < 0,05, baseado no modelo de regressão de Cox, ajustado por todas as variáveis descritas na tabela.

Resíduos de Schoenfeld: * p = 0,651; ** p = 0,367, estratificado pela variável circunferência da cintura.

A partir desse modelo ajustado, testes de interação multiplicativa do controle glicêmico com as covariáveis foram analisados. Contudo, os resultados demostraram uma amplitude muito grande dos IC95%, sugerindo imprecisão dos resultados. Dessa forma, estes resultados não foram apresentados no presente trabalho. Adicionalmente, considerando que a análise descritiva dos grupos de diabetes mellitus mostrou diferença significativa em relação ao tempo de diagnóstico de diabetes mellitus, foram estimados modelos de regressão de Cox apenas para os indivíduos com diabetes mellitus, como uma análise de sensibilidade. No modelo bruto, o risco de morte foi semelhante entre o grupo diabetes mellitus com controle glicêmico inadequado em relação ao grupo diabetes mellitus com controle glicêmico adequado (HR = 1,01; IC95%: 0,98-1,05). Não foi possível estimar o modelo ajustado devido ao pequeno número de participantes com diabetes mellitus distribuídos nos grupos diabetes mellitus controle adequado e diabetes mellitus com controle inadequado. 

**Figura 2:**
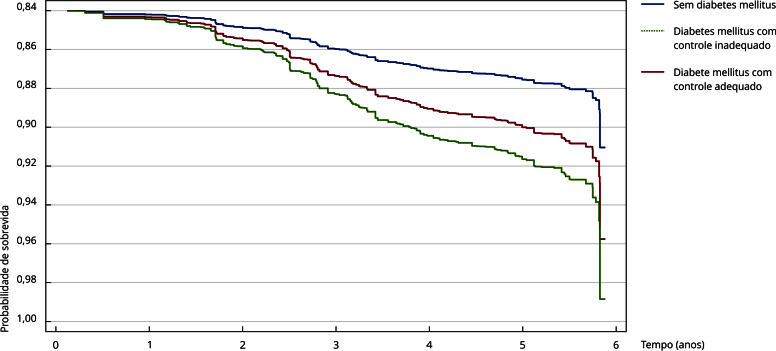
Sobrevida em cinco anos baseado no modelo de regressão de Cox, ajustado por sexo, faixa etária, escolaridade, área de residência, consumo de frutas e vegetais, tabagismo, consumo excessivo de álcool, prática de atividade física, doenças do coração e hipertensão arterial sistêmica. *Estudo Longitudinal da Saúde dos Idosos Brasileiros* (ELSI-Brasil), 2015-2021.

Por fim, de acordo com o modelo ajustado estratificado pela circunferência da cintura, a probabilidade de sobrevida das categorias de diabetes mellitus foi plotada para a média de 5 anos de seguimento. Observou-se um padrão semelhante dessas curvas para todos os grupos de diabetes mellitus (sem diabetes mellitus, diabetes mellitus com controle glicêmico adequado e diabetes mellitus com controle glicêmico inadequado), com uma diminuição mais acentuada da sobrevida a partir do terceiro ano. A probabilidade de sobrevida foi menor entre os adultos mais velhos com diabetes mellitus com controle glicêmico inadequado (p = 0,008) (Figura 2).

## Discussão

Os resultados mostraram que entre os adultos mais velhos brasileiros comunitários, com 50 anos ou mais, a prevalência de diabetes mellitus foi de 16%, dos quais 49,1% apresentaram controle glicêmico inadequado e maior duração da doença, com média de 10,3 anos desde o diagnóstico. O principal resultado foi que o risco de morte por todas as causas foi maior nos indivíduos com diabetes mellitus com controle glicêmico inadequado quando comparados àqueles sem diabetes mellitus. O risco de morte nos indivíduos com controle glicêmico adequado do diabetes mellitus não diferiu estatisticamente do risco de morte dos indivíduos sem diabetes mellitus. Entretanto, deve-se considerar que o tamanho reduzido da amostra nesse subgrupo pode ter resultado em menor precisão das estimativas, de modo que a ausência de significância estatística não deve ser interpretada como ausência de risco. Além disso, o sexo masculino, a faixa etária mais avançada, o consumo excessivo de álcool e a presença de doenças do coração (infarto do coração, angina do peito ou insuficiência cardíaca) identificaram aqueles com maior risco de mortalidade. 

A prevalência de diabetes mellitus nesse estudo foi semelhante à prevalência encontrada na *Pesquisa Nacional de Saúde* (PNS) de 2019 com brasileiros de 60 anos ou mais ^4^. Também houve semelhança na fração que atingiu a meta glicêmica (50,9%) com a fração encontrada na PNS (2013) com adultos brasileiros de 18 anos ou mais (48,1%) ^12^. Destaca-se ainda que o tempo desde o diagnóstico de diabetes mellitus foi maior entre aqueles que tinham o controle glicêmico inadequado, corroborando achados de estudos prévios conduzidos na Dinamarca e nos Estados Unidos ^16^ ^,^ ^19^. 

Estudos internacionais mostram que manter níveis adequados de HbA1c em pessoas idosas com diabetes mellitus é um desafio comum. Na *Pesquisa Nacional de Saúde e Nutrição* (NHANES, acrônimo em inglês) dos Estados Unidos, praticamente 50% dos indivíduos com diabetes mellitus diagnosticado e com idade ≥ 65 anos atingiram o valor de HbA1c < 7% ^13^ e na *Pesquisas Nacionais de Saúde e Nutrição* do Japão esse valor foi apenas 36% ^33^. Nos países da América Latina, o controle glicêmico inferior a 7mmol/L (6,5%) entre as pessoas idosas (≥ 65 anos) foi de 37,2% no Peru, 43,9% na Venezuela e 41,5% no México urbano ^34^. Esses achados, somados aos resultados deste estudo, apontam para a baixa taxa de controle glicêmico em adultos mais velhos e reforçam a necessidade de estratégias específicas para esse grupo populacional.

Não existe consenso sobre a meta de HbA1c para pessoas mais velhas. Para pessoas idosas saudáveis, preconizam-se valores semelhantes aos recomendados para adultos mais jovens, enquanto para pessoas idosas com fragilidade, com incapacidade funcional ou clinicamente complexas, estudos sugerem metas menos rígidas (≤ 8%-9%). Essa falta de individualização das metas no país pode dificultar a identificação dos grupos mais vulneráveis em relação ao controle glicêmico ^35^. Sabe-se ainda que a idade é um preditor independente para o curso clínico do diabetes mellitus, assim como a duração do diabetes mellitus. Por conseguinte, com a idade avançada, quanto maior for o tempo de evolução do diabetes mellitus, maior o risco de morte por todas as causas ^16^ ^,^ ^18^ ^,^ ^19^. 

O aumento da mortalidade observado entre indivíduos com diabetes mellitus e com controle glicêmico inadequado é coerente com a literatura ^13^ ^,^ ^14^ ^,^ ^15^ ^,^ ^16^ ^,^ ^36^ e pode estar relacionado principalmente ao risco cardiovascular. A hiperglicemia crônica é um fator central no desenvolvimento de complicações cardiovasculares ^37^ ^,^ ^38^ que representam a principal causa de morte em pessoas com diabetes mellitus ^39^. Adultos com diabetes mellitus apresentam risco cardiovascular de 2 a 4 vezes maior do que aqueles sem a doença, e esse risco aumenta com o pior controle glicêmico ^40^, independentemente da presença de outros fatores de risco ^41^ ^,^ ^42^. Murray et al. ^43^, baseados nos efeitos legados do DCCT e *Estudo Prospectivo sobre Diabetes* (UKPDS, acrônimo em inglês) do Reino Unido, mostraram que há uma relação da hiperglicemia com excesso de espécies reativas de oxigênio nas mitocôndrias endoteliais. Essa abundância danifica o endotélio, levando à ativação de vias inflamatórias e pró-trombóticas. Paralelamente, ocorre o acúmulo de produtos de glicação avançada, que modificam proteínas estruturais e regulatórias, além de ativarem receptores que amplificam respostas inflamatórias e vasculares ^43^. 

Contudo, mecanismos cardiovasculares podem explicar a associação independente entre controle glicêmico e risco cardiovascular, mas podem ser insuficientes para explicar toda a diferença de mortalidade geral entre os grupos de controle glicêmico do diabetes mellitus. No presente estudo, descritivamente, a causa básica do óbito por doenças do aparelho circulatório foi semelhante entre esses grupos (p = 0,305), apesar da mortalidade ser maior em adultos mais velhos com doenças do coração (p = 0,004). Dessa forma, outros fatores em comum podem atuar nessa associação.

Por exemplo, a associação entre diabetes mellitus e doença cardiovascular pode ser intensificada por comportamentos de risco modificáveis, como consumo excessivo de álcool, tabagismo, sedentarismo e alimentação inadequada ^1^ ^,^ ^44^. Há evidência de que os indivíduos com diabetes mellitus apresentam maior probabilidade de retomar ao sedentarismo mesmo após iniciar a prática de atividade física. Esse comportamento pode, paradoxalmente, explicar porque o grupo diabetes mellitus com controle glicêmico inadequado tenha relatado maior prática de atividade física como parte do tratamento para o diabetes mellitus, e maior tempo médio de diagnóstico. Tal achado aponta para a necessidade de estratégias mais precoces para a adoção e manutenção de uma vida ativa em pessoas com diabetes mellitus ^45^.

Além disso, no presente estudo, o consumo excessivo de álcool foi um fator significativamente associado à mortalidade. Esse achado deve ser interpretado com cautela, uma vez que a baixa prevalência desse comportamento (6,3%) pode ter reduzido a precisão das estimativas. Sabe-se que o álcool, além de aumentar o risco de desenvolver diabetes mellitus, também dificulta o controle glicêmico e contribui para doenças cardiovasculares, sendo responsável por mais de 200 condições clínicas ^46^ ^,^ ^47^ ^,^ ^48^. Mecanismos propostos incluem o dano pancreático direto ^49^, o ganho de peso e a obesidade induzidos pelo consumo crônico de bebidas alcoólicas ^49^ ^,^ ^50^. Além disso, estudos mostram que medidas de gordura central, como a circunferência da cintura, estão associadas ao pior controle de HbA1c em populações com diabetes mellitus e preveem maior risco de eventos cardiovasculares e mortalidade por todas as causas. Análises têm demonstrado que cada aumento na gordura abdominal está correlacionado a pior desempenho glicêmico e que incrementos na circunferência da cintura estão associados a aumento do risco de mortalidade ^51^. Entretanto, o risco direto de mortalidade associado à circunferência da cintura não pode ser estimado no presente estudo.

A redução da mortalidade prematura por diabetes mellitus depende de múltiplas estratégias, incluindo prevenção do aparecimento da doença, mudanças no estilo de vida, monitoramento clínico e adesão terapêutica ^52^. Essas estratégias são fundamentais para a promoção da qualidade de vida a nível individual, para impedir a sobrecarga dos serviços de saúde e diminuir custos de saúde ^53^. Neste contexto, a HbA1c destaca-se como um marcador confiável do controle glicêmico, com vantagens como baixa variabilidade e não exigência de jejum. A realização rotineira desse exame é recomendada duas vezes ao ano nos protocolos clínicos brasileiros para o tratamento do diabetes mellitus ^54^. A literatura já mostra que cada redução de 1% na HbA1c é associada a uma redução de 7,6% no risco de mortalidade por todas as causas ^55^.

Para esse manejo da HbA1c, a atenção primária à saúde (APS) segue uma estratégia de incentivo ao hábito de vida saudável. No entanto, na prática, as ações ainda se concentram predominantemente no fornecimento de medicamentos. Essa abordagem restrita dificulta a compreensão das necessidades de saúde e limita a efetividade das intervenções. Evidências indicam que a falha no atributo da longitudinalidade da APS, além de dificultar o vínculo entre pessoas com diabetes mellitus e as equipes de saúde, ainda pode dificultar a adesão ao tratamento medicamentoso e não medicamentoso ^12^. 

Este estudo apresenta algumas limitações. O diagnóstico de diabetes mellitus foi baseado em autorrelato, o que pode subestimar a prevalência real da doença, além de estar condicionado ao acesso prévio aos serviços de saúde, sendo que esse acesso prévio pode ser diferencial para algumas características sociodemográficas, como o sexo. O consumo de frutas e vegetais foi avaliado por frequência de consumo, e não por porções padronizadas, o que pode reduzir a precisão da medida ^56^. A variável circunferência da cintura violou a suposição de riscos proporcionais no modelo de Cox, sendo considerada apenas como estrato na análise, o que impediu sua avaliação direta como fator de risco. Por fim, o uso de medida objetiva sanguínea traz ineditismo ao estudo, mas limita seu poder estatístico em explorar a associação entre controle glicêmico e mortalidade estratificada por grupos, como nas diferentes regiões brasileiras ou por causas básicas do óbito. Estudos futuros, com maior poder estatístico, poderiam analisar tais associações.

Como pontos fortes do estudo, destaca-se a amostra representativa de adultos mais velhos comunitários e não amostras hospitalares ou ambulatoriais. O rigor metodológico dos fatores examinados no ELSI-Brasil, suas medidas laboratoriais padronizadas e a representatividade nacional permitiram a investigação adequada de associações epidemiologicamente relevantes. Ainda, o uso da *linkagem* com o SIM permitiu confirmar quase 90% dos óbitos informados e 10% das perdas, diminuindo possíveis vieses de seleção ^23^.

Em síntese, os resultados evidenciam que o controle glicêmico inadequado está fortemente associado à mortalidade por todas as causas em adultos mais velhos com diabetes mellitus. Tais achados reforçam a necessidade de políticas públicas que ampliem o acesso ao diagnóstico, ao monitoramento regular da HbA1c e às intervenções integrais no SUS, especialmente na APS e para os subgrupos de maior risco: adultos mais velhos do sexo masculino, indivíduos com histórico de consumo excessivo de álcool e aqueles com doenças do coração.

## Data Availability

Os dados de pesquisa estão disponíveis mediante solicitação à autora de correspondência.
